# Schizophrenia and pain: a prospective clinical study from 2002-2022 on 753 men with schizophrenia and sensitivity to pain compatible with the cause, reduction or lack of sensitivity

**DOI:** 10.1192/j.eurpsy.2025.1203

**Published:** 2025-08-26

**Authors:** E. Guertzenstein

**Affiliations:** Dep. Neurologia - Divisão de Neurocirurgia, Faculdade de Medicina da Universidade de S. Paulo, S. Paulo, Brazil

## Abstract

**Introduction:**

Schizophrenia is a complex neuropsychiatric disorder, which affects 1% of people in the world. It presents marked heterogeneity in terms of clinical presentation, course and prognosis, although patient with schizophrenia may have diseases of other physical causes, in which pain has a protective role for the individual and diagnostic importance. Studies on pain in schizophrenia are rare and predominantly experimental. The cause of decreased sensitivity or lack of sensitivity to pain in patients with schizophrenia is unknown. Among the hypothesis are lesions in the thalamus and psychotic symptoms that would decrease the patient’s concentration or divert his attention from the pain.

**Objectives:**

The aim of this research was to study the presence/absence and intensity of pain compatible with the cause in 753 men that we have been treating for schizophrenia for 20 years.

**Methods:**

We asked all our patients with schizophrenia at their first consultation in 2002 whether they would be willing to be clinically investigated for the presence/absence and intensity of pain compatible with any cause.

We divided our patients into 3 groups:

Group 1: 51 patients aged 17 – 25 years

First episode currently symptomatic-negative symptoms.

Group 2: 325 patients aged 27-31 years

Multiple episodes currently symptomatic, 25 negative symptoms, 300 positive symptoms.

Group 3: 377 patients aged 40-45 years

Continuous currently – 7 negative symptoms, 370 positive symptoms.

These patients were examined every 3 months by clinicians and dentists for 20 years.

Treatment

Patients with:

- Positive symptoms (delusional beliefs, hallucinatory perception, and disorganization of thinking and behavior) associated with excessive dopamine release, who respond adequately to (FL-APM) First Line dopamine antagonist medication.
- Positive symptoms which does not respond adequately to FL-APM, who respond adequately to chlozapine.
- Refractory schizophrenia treated with electroconvulsive therapy.

**Results:**

100 patients quit of the survey

653 had (at least once in these 20 years) headache, toothache or earache.

51 did not complain of pain with trigeminal neuralgia.

30 patients did not complain of pain when they had herpes zoster.

Patients with negative schizophrenic symptoms and resistance to treatments showed an absence of pain (pertinent to their physical illness)

**Conclusions:**

The causes of reduced or absent pain sensation in patients with schizophrenia are unknown. Proposed explanations include thalamic damage and psychotic symptoms that render the patient unable to perceive pain. Our patients did not have thalamic damage but had severe negative psychotic symptoms and resistance to treatments.

**Disclosure of Interest:**

None Declared

